# Progesterone and brassinosteroids synergistically enhance progesterone removal and antioxidant capacity of *Solanum nigrum* L.

**DOI:** 10.1007/s12298-024-01496-9

**Published:** 2024-08-02

**Authors:** Ana Pinto, Daniela Correia da Silva, Ana Cardoso, Fátima Fernandes, Cristiano Soares, Patrícia Valentão, Fernanda Fidalgo, Jorge Teixeira

**Affiliations:** 1 GreenUPorto – Centro de Investigação em Produção Agroalimentar Sustentável – Inov4Agro & Departamento de Biologia, Faculdade de Ciências, Universidade do Porto, Porto, Portugal; 2 REQUIMTE/Laboratório de Farmacognosia, Departamento de Química, Faculdade de Farmácia, Universidade do Porto, Porto, Portugal; 3 Dep° de Biologia, Faculdade de Ciências da Universidade do Porto, Edifício FC4. Rua do Campo Alegre, S/N. 4169-007, Porto, Portugal

**Keywords:** Animal sex hormones, Environmental clean-up, Gene expression, Oxidative stress, Black nightshade

## Abstract

Progesterone (PROG) has been detected at various concentrations in the environment and has adverse effects on humans and wildlife. This work evaluated the impact of PROG in *Solanum nigrum* L. plants, its removal capacity, and how 2,4-epibrassinolide (24-EBL) affects this process. Three treatments were used: (1) control, (2) irrigation with 0.8 µM PROG, and (3) treatment with 0.8 µM PROG after a pre-treatment with a foliar application of 1 µM 2,4-EBL (PROG/24EBL). After 20 days of treatment, no PROG was detected in the nutrient solution or plant tissues, indicating that the PROG was removed and metabolized. Lipid peroxidation significantly decreased in response to PROG in shoots and roots, and this effect was even more significant for both organs of the PROG/24EBL plants. Additionally, both treatments in both organs showed a decrease in H_2_O_2_ levels, and both steroid hormones increased the plants’ antioxidant system at both the biochemical and gene expression levels. In conclusion, *S. nigrum* can swiftly remove PROG without affecting its growth, and the use of 24-EBL synergistically decreases oxidative damage by increasing the activity of the antioxidant system and enhancing plant PROG removal ability.

## Introduction

Steroid hormones such as estrogens, progesterone, and testosterone are, by definition, crucial substances for the proper function of organisms, mediating a wide variety of vital physiological functions, such as sexual differentiation, maintenance of cellular homeostasis, and regulation of events associated with growth (Janeczko 2021). In addition to their natural excretion by animals, but mainly due to the widespread use of birth-control pills and other analogous drugs used for the treatment of hormonal disorders, steroid sex hormones have been detected in wastewater effluents and surface water. Consequently, they accumulate in the biosphere, and progesterone concentrations ranging from 0.59 to 2.33 µg mL^−1^ have been reported to accumulate in several influents of water-treatment plants in southeastern Finland (Sirén and El Fellah 2016) and 10 to 25 µg kg^−1^ agricultural soils in Malaysia (Ho et al. 2014).

The presence of natural and synthetic steroids in the environment has generated significant concern among the scientific community and the general public since these compounds are endocrine-disrupting chemicals (EDCs) (Rocha and Rocha 2022). The harmful effects of EDCs occur through mimicking or simulating natural hormones, changing the standard pattern of organism biosynthesis and metabolism, as well as hormone receptor levels. Previous studies have indicated that human health can be affected by EDCs, increasing the chance of incidence of breast cancer, endometriosis, and testicular and prostate cancers and reducing male fertility (Nohynek et al. 2013; Rocha and Rocha 2022; Solomon and Schettler 2000). In addition to the potential effects on human health, several studies suggest that synthetic androgens and progestins can disrupt normal endocrine function in many aquatic organisms, causing adverse effects on fish reproduction, even at low ng L^−1^ (Rocha and Rocha 2022; Söffker and Tyler 2012; Zeilinger et al. 2009).

Nonetheless, additional studies are needed to better understand these steroid hormones' biological impact on plants. *Solanum nigrum* L., or black nightshade, as it is commonly known, can degrade and/or accumulate both organic and nonorganic pollutants (Alves et al. 2023; de Sousa et al. 2017, 2013; Ferraz et al. 2012; Fidalgo et al. 2013, 2011; Kucerová et al. 2000; Moreira et al. 2018; Pinto et al. 2023; Soares et al. 2016; Sousa et al. 2020b; Teixeira et al. 2011, 2013, 2015), revealing that this sturdy species is suitable for such studies.

Plants are commonly exposed to several biotic and abiotic stress factors that lead to the overproduction of reactive oxygen species (ROS), such as hydroxyl radicals (^.^OH), superoxide anion (O_2_^.−^), and hydrogen peroxide (H_2_O_2_). In excess, ROS can trigger cellular damage by oxidizing membrane lipids, affecting membrane permeability and structural integrity, and through direct interactions with various macromolecules (proteins, DNA, etc.). In plants, the toxic effects of ROS can be counteracted by nonenzymatic and enzymatic antioxidants. A standard adaptive response of plants to oxidative stress is the increase in antioxidant compound levels and the increase in the activity and/or expression of one or more antioxidant enzymes (Soares et al. 2019).

ROS detoxification pathways result in high GSH:GSSG ratios and the participation of related enzymes, such as Glutathione Reductase (GR, EC 1.8.1.7), Glutathione *S-*Transferase (GST, EC 2.5.1.18), and glutathione peroxidase (GPX, EC 1.11.1.9), which play indispensable roles in the detoxification of ROS (Nogueirol et al. 2016). Reduced glutathione (GSH) is considered a major intracellular redox buffer in plants. Increased activities of the GSH biosynthesis and regeneration enzymes and maintaining a high GSH:GSSG ratio enhance plants' resistance and adaptation to various environmental conditions (Labrou et al. 2015; Rabêlo et al. 2017; Soares et al. 2019). In fact, broccoli seed priming with GSH improved growth traits, total soluble proteins, chlorophyll content, mineral content, and gas exchange parameters of Pb-exposed seedlings (Ahmad et al. 2023), further demonstrating the importance of GSH in response to abiotic stress.

Several phytohormones, such as salicylic acid (SA) (Hayat et al. 2010) and brassinosteroids (BRs), have been implicated in modulating plant responses to oxidative stress (Bari and Jones 2009; Fariduddin et al. 2014; Sousa et al. 2020b) by acting with the antioxidant system. BRs are steroidal plant growth regulators distributed ubiquitously throughout the plant kingdom and are well known to play a critical role in plant growth, development, and seed germination. It has also been demonstrated that applying BRs has specific antioxidant effects against environmental stresses. In fact, the exogenous application of 24-epibrassinolide (24-EBL), a class of BR, detoxifies the stress generated by NaCl in *Brassica juncea* (Ali et al. 2008) and by Ni (Soares et al. 2016) and Zn in *S. nigrum* (Sousa et al. 2020b) by increasing antioxidant responses.

To date, very few reports have been published regarding the actions of progesterone in plants (Janeczko 2021). PROG increased seed germination and plant defense against abiotic and biotic stress by stimulating the activity of antioxidant enzymes (Genisel et al. 2013). The addition of PROG mitigated cold stress in maize seedlings by activating the cytochrome pathway, especially the alternative respiratory pathway (Erdal and Genisel 2016). However, most of the present investigations on progesterone in plants are limited to the physiological level, and additional investigations at the molecular level are needed to elucidate PROG signaling pathways (Li et al. 2022).

Bearing in mind the important role of the antioxidant system in plant tolerance against several kinds of stresses and the protective effects of BRs, the main goal of this study was to evaluate the impact of PROG on the physiological responses of *S. nigrum* and its ability to remove this hormone, as well as to determine whether a pre-treatment with 24-EBL would enhance the removal capacity of this species. For that purpose, growth parameters, photosynthetic pigments, several enzymatic and nonenzymatic antioxidant endpoints, and oxidative stress markers were analyzed both at the biochemical and molecular levels, as was the determination of PROG levels in the nutrient solutions and in plant tissues after 20 days of exposure.

## Materials and methods

### Growth conditions and biometric analysis

Seeds of *S. nigrum* were collected from plants grown at Foz do Douro, Porto, Portugal (41°09′17,80’’N and 8°39′47,91’’O). Seed disinfection, stratification, and germination were performed as previously described (Moreira et al. 2018). Seedlings with similar development were selected and transplanted into plastic pots with expanded vermiculite and perlite (2:1) as root substrate. These were grown in a growth chamber at 24 °C, with Hoagland solution (HS) being supplied when needed, under a 16 h/8 h day/night photoperiod with a photosynthetically active radiation of 120 mmol m^−2^ s^−1^ until seedlings reached 1.5 cm tall, at which point they were divided in the following groups: 1) Control—without any phytohormone supplied and watered with HS; 2) PROG—exposure to 0.8 µM (251.6 µg L^−1^) progesterone (water-soluble, HBC complex, Merck Portugal, dissolved in the HS), to simulate a soil contaminated with this hormone based on data previously reported (Rocha and Rocha 2022); 3) PROG/24-EBL—plants also exposed to 0.8 µM (251.6 µg L^−1^) PROG in HS but pre-treated with a single foliar application of 1 µM 24-epibrassinolide (24-EBL; dissolved in a small amount of ethanol and the final volume completed with deionized water) prior to PROG exposure; the choice for using 1 µM 24-epibrassinolide was based on previous reports where it was shown that a pre-treatment with 1 µM 24-epibrassinolide increased *S. nigrum* tolerance to Ni (Soares et al. 2016) and Zn (Sousa et al. 2020b). Whenever the HS solutions ran low, the remaining solution was discarded and replaced by a new one.

A “no plants” approach was also used to determine whether PROG removal could be due to non-plant-related factors, such as microbiological degradation or adsorption to the vermiculite:perlite matrix. For this purpose, samples for PROG quantification in the HS solution were collected before replacement with a new solution, along with samples from the two treatments (see below).

At the end of the treatment period (20 days after exposure), the shoots and roots of at least three plants from each condition were used for the determination of several growth parameters: length (cm), fresh weight (g), and dry weight (g). Additionally, shoots and roots (n ≥ 3) were ground to a fine powder in liquid nitrogen and stored at -80 °C for later biochemical and molecular studies and PROG quantification. HS from “no plants”, PROG and PROG/24-EBL treatments were collected throughout 6 days (days 10 to 15) before the solution was discarded and 20 min after its replacement, and at the end of the treatment (day 20) for PROG determination by HPLC–DAD.

### Progesterone determination

#### Standards and reagents

Progesterone (a water-soluble HBC complex) was purchased from Merck. Potassium hydroxide was acquired from Pronalab (Lisboa, Portugal), hexane from Merck, and ethanol and anhydrous sodium sulfate from Panreac (Castellar del Valle`s, Spain). HPLC-grade methanol and acetonitrile were obtained from Merck. Water was deionized using a Milli-Q water purification system (Millipore, Bedford, MA, USA).

#### Hydrolysis and HPLC–DAD analysis

A complex of the hormone with methyl-ß-cyclodextrin was acquired to increase the solubility of PROG in water. A purification step was performed with an organic solvent from 2 mL samples of Hoagland’s solution supplemented with PROG: the solutions were transferred to a separation funnel to extract the lipophilic components with 4 mL of n-hexane 3 times. The organic phase was dehydrated with anhydrous sodium sulfate and evaporated to dryness under reduced pressure at 30 °C. The residue was redissolved in acetonitrile:methanol (70/30, v/v) and filtered through a 0.45 µm membrane (Millipore). Tissues from each treatment (ca. 5 g) were extracted with 100 mL of 80% MeOH for 2 h under stirring (300 rpm). The extract was centrifuged (10 min, 4,000 xg), and the material was re-extracted with 100 mL of 80% MeOH (15 min). The supernatants were evaporated to dryness under reduced pressure at 30 °C. The residue was dissolved in acetonitrile:methanol (70/30, v/v) and filtered through a 0.45 µm membrane (Millipore).

Twenty microliters of each extract were analyzed at room temperature in an HPLC unit (Gilson) using a reversed-phase Hypersil ODS (20 × 0.4 cm i.d. × 5 μm) column. The mobile phase was methanol:acetonitrile (30/70, v/v) at a flow rate of 0.8 mL min^−1^. Detection was achieved with a Gilson diode array detector (DAD). Spectral data from all the peaks were accumulated in the 190–400 nm range. The data were processed with an Unipoint system Software (Gilson Medical Electronics, Villiers le Bel, France). The peak purity was checked by software. PROG was identified by comparison of its retention time and UV absorption spectrum with those of an authentic standard. Quantification was achieved by the absorbance recorded in the chromatograms registered at 240 nm. The calibration curve (y = 1,152,372.5574*x*) (r^2^ = 0.9971) was obtained with standard solutions (at seven different concentrations) prepared according to the conditions described above.

### Chlorophyll and carotenoid contents

Photosynthetic pigments were extracted and quantified using the procedure of Lichtenthaler (1987) and expressed as mg g^−1^ fresh weight (f.w.).

### Determination of H_2_O_2_, lipid peroxidation, and proline concentrations

Hydrogen peroxide was extracted from 0.3 g of frozen tissue (shoots or roots) to 1 mL of 0.1% (w/v) trichloroacetic acid (TCA), and the amount of H_2_O_2_ was calculated using ε = 0.28 µM^−1^ cm^−1^ (Alexieva et al. 2001). The results are expressed as pmol g^−1^ f.w.

Thiobarbituric acid reacting substances (TBARS), an indicator of membrane lipid peroxidation, were measured in terms of malondialdehyde (MDA) content as described previously (Heath and Packer 1968). A molar extinction coefficient of 155 mM^−1^ cm^−1^ was used, and the results are expressed as nmol MDA g^−1^ f.w.

The shoots and roots' proline concentrations were determined via a ninhydrin-based colorimetric assay (Bates et al. 1973). The results are expressed as µg proline g^−1^ f.w.

### Glutathione (GSH) determination

GSH extraction was accomplished by homogenizing 0.5 g of plant tissue with 2.5 mL of 3% (w/v) sulfosalicylic acid. After 10 min of incubation at 4 °C, the homogenate was centrifuged at 10,000 xg for 10 min at 4 °C. GSH levels were determined by quantifying the amount of DTNB (5,5’-dithiobis [2-nitrobenzoic acid]) directly reduced by GSH at 412 nm. For that purpose, 750 µL of a working solution [1 mM EDTA in 100 mM phosphate buffer (pH 7.0) and 400 µL of DTNB (1.5 mg mL^−1^; dissolved in DMSO] were added to 50 µL of extract and H_2_O in a final volume of 1 mL. For the blank, 3% (w/v) sulfosalicylic acid was used instead of extract. For the standard curve, known GSH concentrations were used instead of the extract. The GSH concentrations are expressed as the nmol of GSH g^−1^ f.w.

### Ascorbate peroxidase (APX), glutathione S-transferase (GST), gamma-glutamylcysteine synthetase (ɣ-ECS), and glutathione reductase (GR) activity determinations

APX was extracted from the roots and shoots according to Fidalgo et al. (2011). GST and ɣ-ECS were extracted and quantified as described previously Sousa et al. (2021). GR activity was determined following the oxidation of NADPH at 340 nm using the extinction coefficient of 6.22 mM^−1^ cm^−1^ (Yannarelli et al. 2007). The activities of these enzymes were expressed in nmol min^−1^ mg^−1^ protein.

For all enzymes, the protein content of the extracts was measured according to Bradford (1976) using BSA as a standard.

### Statistical analysis

All assays and measurements were performed at least in triplicate (n ≥ 3). Variance analysis was performed using Fisher’s test, and the means were statistically analyzed using a two-sided Student’s t-test. *p* < 0.05 was assumed to indicate statistical significance.

### RNA extraction and semiquantitative RT‒PCR

Total RNA was extracted using TRIzol reagent (Invitrogen, USA) according to the manufacturer’s instructions. The RT‒PCRs consisted of two steps: reverse transcription (RT) (using the primer R9 – 5’-TTT TTT TTT TTT TCG AAC TCG AGC TCA GGA GCA GTG AGA CGA GTG ACC-3’) (Fidalgo et al. 2011), followed by cDNA-specific PCR amplification. The RT reactions were performed using NZY M-MuLV Reverse Transcriptase (NZYTech®, Portugal) according to the recommended protocol.

The specific semiquantitative PCR evaluations were performed at least in triplicate in a final volume of 20 µL with Kapa Taq ReadyMix (Kapa Biosystems, UK) and assembled with 1 µL of a 1:10 dilution of the RT reaction as a template. The primer sets and programs used to amplify APX and tubulin (Tub; used as loading control) were described previously (Fidalgo et al. 2011). The sequences used to amplify GST and ɣ-ECSs were previously published (Martins and Teixeira 2021; Teixeira et al. 2015). Because there was only one (incomplete) cDNA nucleotide sequence encoding *S. nigrum*’s GR, the primer sets used for the detection of GR transcripts were designed based on the alignment of several GR-encoding sequences from other *Solanum* species in *PrimerIdent* (Pessoa et al. 2010), resulting in the following sequences (forward/reverse): 5’-TGG TGA ACT TGA GGA TGC-3’/5’-CAA CTA GTG CCC TCA TTT CG-3’. The GR-related PCRs were performed on an S-96 thermocycler (Quanta Biotech, UK) with the following program: 94 °C-2’, followed by 28 cycles of 94 °C-30″, 50 °C-30″ and 72 °C-1’. The final extension was performed at 72 °C for 5 min, and the reactions were stored at 4 °C until analysis by agarose gel electrophoresis.

The PCR products were loaded into 0.8% (w/v) agarose gels, and DNA electrophoresis was performed according to standard molecular biology procedures. Subsequent electrophoresis using the same PCR products was performed with adjusted volumes, considering those needed to observe uniform bands for the tubulin amplicon in all wells/situations to obtain semiquantitative results for the studied genes (Teixeira et al. 2015). All gel images were acquired with a ChemiDoc™ MP System (Bio-Rad Laboratories, USA).

## Results

### Growth parameters

No significant differences were observed in the biometry of *S. nigrum* plants from both treatments. Additionally, PROG- and PROG/24-EBL-treated plants did not show any signs of toxicity (data not shown).

### Progesterone quantification

**Fig. 1:**
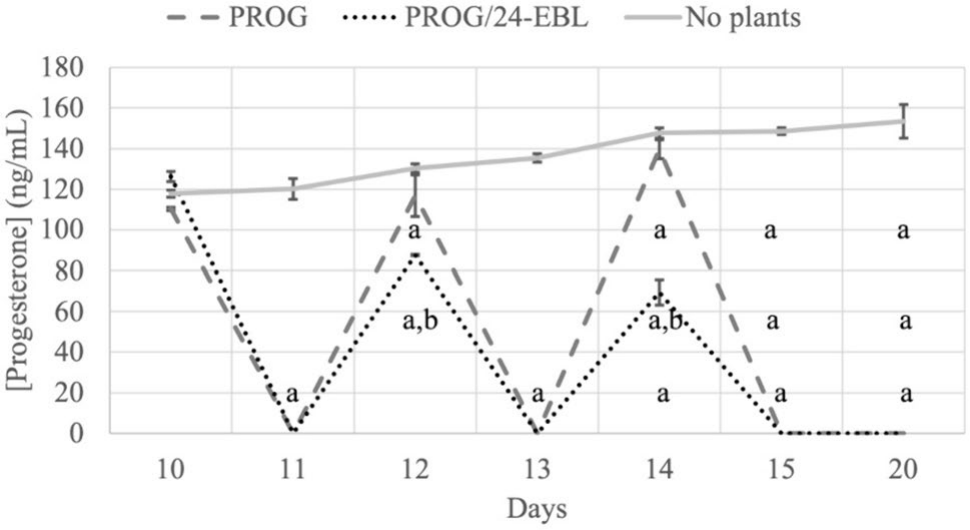
Progesterone levels quantified in the nutrient solutions added to the plants. On days 10, 12, and 14, aliquots were retrieved 20 min after adding the PROG-containing nutrient solution. Aliquots were obtained on days 11, 13, and 15 before adding a fresh PROG-containing nutrient solution. PROG—plants exposed to 0.8 µM (251.6 µg L^−1^) PROG; PROG/24-EBL—plants exposed to 0.8 µM PROG but pre-treated with a foliar application of 1 µM 24-epibrassinolide; “No plants” – root support (vermiculite:perlite) watered with HS containing 0.8 µM (251.6 µg L^−1^) PROG. Data presented are mean ± SD (n ≥ 3). ^a^Significant difference from “No plants” at *p* < 0.05. ^b^Significant difference from PROG at *p* < 0.05

The HPLC–DAD analysis of the hydrolyzed commercial standard showed progesterone eluting at 2.8 min at 240 nm (λmax), as previously reported (Almeida and Nogueira 2006). By a 6-day period (days 10 to 15) evaluation of the PROG removal by the plants, it was possible to observe that PROG levels in the nutrient solution were reduced to nondetectable levels after one day of exposure, independently of the plants being pre-treated or not with 24-EBL (Fig. 1). Moreover, at the end of the experiment, no PROG was detected in all analyzed samples (plant tissues (data not shown), and nutrient solutions – Fig. 1). Considering the “No plants” situation, PROG levels remained always high throughout the experimental period, with a slight, but not statistically significant, increase throughout time.

The pre-treatment with 24-EBL resulted in a significant reduction in PROG levels in the solution compared to the PROG treatment, by 1.33- and twofold, on days 11 and 14, respectively.

### Biochemical determinations

#### Total chlorophyll and carotenoid content

Chlorophyll levels (Chl a and Chl b) and carotenoids in the shoots of plants exposed to PROG and to PROG/24-EBL were similar to those of control plants (Table 1).

**Table 1:** Effect of PROG (0.8 µM) and PROG (0.8 µM)/24-EBL (1 µM) on chlorophyll (a + b) and carotenoids contents (mg g^−1^ f.w.), H_2_O_2_ (pmol g^−1^ f.w.), MDA levels (nmol g^−1^ f.w.), proline (µg g^−1^ f.w.) and GSH contents (nmol g^−1^ f.w.) in shoots and roots of *S. nigrum* L. plants after 20 days of treatment

Parameters	Control	PROG	PROG/24-EBL
Shoots			
Total chlorophyll	1.35 ± 0.13	1.31 ± 0.04	1.33 ± 0.07
Carotenoids	0.14 ± 0.01	0.15 ± 0.01	0.17 ± 0.02
H_2_O_2_	4.54 ± 0.10	3.36 ± 0.35^a^	2.20 ± 0.13^a,b^
MDA	22.20 ± 0.46	14.76 ± 0.52^a^	7.41 ± 0.60^a,b^
Proline	0.06 ± 0.0025	0.069 ± 0.0023	0.077 ± 0.0026^a,b^
GSH	48.38 ± 0.81	44.31 ± 0.59^a^	37.82 ± 0.29^a,b^
Roots			
H_2_O_2_	1.53 ± 0.06	0.99 ± 0.06^a^	0.70 ± 0.04^a,b^
MDA	11.52 ± 0.49	5.74 ± 0.58^a^	4.19 ± 0.11^a,b^
Proline	0.035 ± 0.0025	0.041 ± 0.002	0.058 ± 0.001^a,b^
GSH	23.78 ± 0.48	19.6 ± 0.97^a^	14.46 ± 0.73^a,b^

Data presented are mean ± SD (n ≥ 3)

^a^Significant difference from control at *p* < 0.05

^b^Significant difference from PROG at *p* < 0.05

#### Hydrogen peroxide quantification

The spectrophotometric detection of shoots’ H_2_O_2_ levels revealed a significant decrease of 26% and 52% for the PROG and PROG/24-EBL treatments, respectively, while significant reductions of 35% and 54% were detected for the roots of those treatments (Table 1).

#### MDA and free proline content

The lipid peroxidation, expressed in terms of MDA levels, suffered a significant decrease in shoots and roots of both treatments (Table 1), with a reduction in shoots of 33.5% and 66.5% in the PROG and PROG/24-EBL treatments, respectively. In contrast, in roots, it decreased 50% and 64% in the PROG and PROG/24-EBL-treated plants, respectively.

As shown in Table 1, proline levels significantly increased only with the PROG/24-EBL treatment: shoots by 1.3-fold and roots by 1.7-fold.

#### Glutathione levels

GSH levels decreased with both treatments, both in shoots and roots (Table 1). It significantly reduced by 9% and 18% in shoots and roots in plants exposed to 0.8 µM of PROG and by 22% and 40% in shoots and roots treated with the two hormones.

#### Antioxidant enzyme activities and transcript accumulation patterns

γ-ECS, APX, GR, and GST activities were determined spectrophotometrically for shoots and roots of *S. nigrum* plants (Table 2). PROG exposure did not alter γ-ECS activity in shoots and roots. However, in the PROG/24-EBL treatment, the activity of this enzyme increased 2.4-fold in shoots and decreased 1.21-fold in roots compared to the control.

**Table 2:** Effect of PROG (0.8 µM) and PROG (0.8 µM)/24-EBL (1 µM) on APX (μmol min^−1^ mg^−1^ prot), GR, γ-ECS and GST activities (nmol min^−1^ mg^−1^ prot) in shoots and roots of *S. nigrum* plants after 20 days of treatment

Parameters	Control	PROG	PROG/24-EBL
Shoots			
APX	9.38 ± 2.05	18.40 ± 0.16^a^	21.10 ± 1.22^a,b^
GR	9.97 ± 0.90	12.67 ± 0.76^a^	24.16 ± 0.56^a,b^
γ-ECS	1.22 ± 0.03	1.18 ± 0.05	2.93 ± 0.01^a,b^
GST	2.83 ± 0.56	1.20 ± 0.46^a^	7.35 ± 0.42^a,b^
Roots			
APX	3.72 ± 0.37	10.50 ± 0.58^a^	13.96 ± 2.10^a^
GR	10.46 ± 0.70	9.63 ± 0.69	5.38 ± 0.63^a,b^
γ-ECS	16.10 ± 0.15	15.55 ± 0.17	13.29 ± 0.25^a,b^
GST	25.96 ± 1.93	14.63 ± 0.94^a^	13.12 ± 5.13^a^

Data presented are mean ± SD (n ≥ 3)

^a^Significant difference from control at *p* < 0.05

^b^Significant difference from PROG at *p* < 0.05

Additionally, two ascorbate–glutathione cycle-related enzymes were studied: APX and GR. In shoots, the activity of both enzymes was significantly increased by both treatments, with PROG/24-EBL-treated plants presenting the highest values. Shoot APX activity increased almost twofold in the PROG treatment and 2.25-fold with both hormones. Root APX activity of the PROG treatment significantly increased 2.8-fold, and a higher increase (3.8-fold) was detected in the PROG/24-EBL-treated plants. Shoot GR activity increased 1.3- and 2.4-fold in the respective treatments. In contrast, root GR activity did not present variations in the PROG-treated plants. It showed a significant 1.9-fold decrease in plants treated with the two hormones compared to the control and a 1.1-fold decrease compared to the PROG-treated plants.

The shoot GST activity significantly decreased 2.4-fold in PROG-treated plants compared to the control but increased 2.6-fold with the PROG/24-EBL treatment. PROG/24-EBL shoot GST activity significantly increased 6.1-fold compared to the PROG treatment. In roots, both treatments lead to significant decreases of GST activity: 1.8- and ≈twofold, PROG and PROG/24-EBL, respectively.

**Fig. 2:**
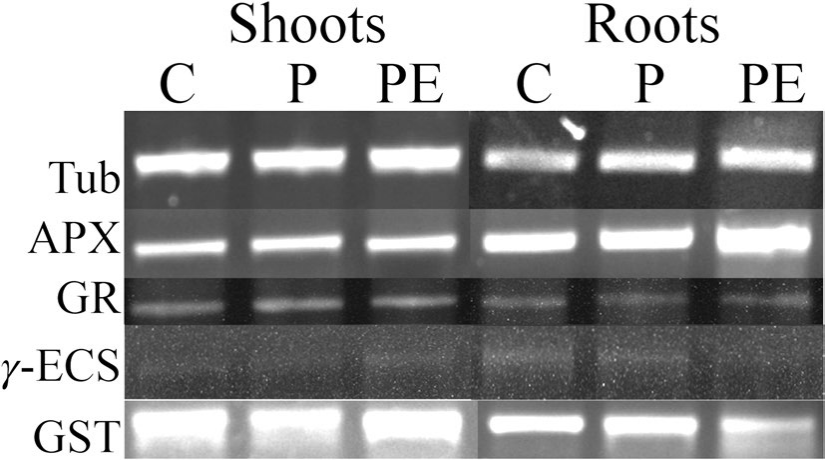
Typical transcript accumulation patterns for the APX-, GR-, γ-ECS- (GSH1), and GST-encoding genes in shoots and roots of *S. nigrum* plants, analyzed by semiquantitative RT-PCR followed by 0.8% (w/v) agarose gel electrophoresis. Tub—tubulin; C—control; P—0.8 µM progesterone; PE—0.8 µM progesterone + 1 µM 24-epibrassinolide

The expression of the genes encoding four antioxidant enzymes was studied in shoots and roots of control, PROG, and PROG/24-EBL-treated plants by semiquantitative RT‒PCR (Fig. 2). The analysis of the GR mRNA accumulation patterns revealed no changes among the three groups of plants and for both organs. Only the plants treated with both hormones increased APX’s mRNA accumulation in roots, and no variation was observed in the other situations.

The levels of γ-ECS mRNA did not change in response to PROG in both shoots and roots, but when the plants were pre-treated with 24-EBL, an increase was observed in shoots, and a decrease was observed in roots. In shoots, PROG negatively affected GST-related transcript accumulation, but an increase was observed with the 24-EBL pre-treatment. Concerning roots, a decrease in GST-encoding mRNA accumulation was observed in plants exposed to both treatments, with PROG/24-EBL presenting the lowest accumulation (Fig. 2).

## Discussion

Plant growth is the result of integrated and regulated physiological processes. To assess the effects of PROG in *S. nigrum* plants, 0.8 µM PROG was added to the nutrient solution. At the same time, another similar set was made, but with a foliar pre-treatment with 24-EBL, to determine if this steroid hormone would enhance this species’ tolerance or removal capacity towards PROG, like in previous studies when this species was exposed to high levels of Ni (Soares et al. 2016) or Zn (Sousa et al. 2020b). Exposure of *S. nigrum* to 0.8 µM PROG did not trigger a reduction of biomass and decreased the physiological stress-related parameters tested (lipid peroxidation and H_2_O_2_). Hence, it can be assumed that *S. nigrum* plants’ growth was not compromised by the PROG concentration used, which corresponds to the average concentration found in PROG-contaminated sites (Rocha and Rocha 2022).

The HPLC–DAD analysis showed that no PROG was detected in the nutrient solutions one day after the nutrient solution was replaced. Still, it always remained high in the “no plants” situation. This shows that: 1) the substrate used did not affect the PROG levels in the nutrient medium; 2) no PROG degradation occurred throughout time by external factors to the plants; 3) the plants were the only responsible factor for the removal of PROG from the solution; 4) and that the PROG removal rate was very high (Fig. 1). Moreover, because the PROG levels on the days when the nutrient solution was added to the tray were consistently higher in the non-pre-treated plants with the 24-EBL situation (i.e., PROG), one can conclude that the 24-EBL pre-treatment boosted this removal capacity. Plus, since no PROG was detected in the nutrient solutions and plant tissues of both treatments at the end of the treatment period, it further supports the idea that the plants examined were able to uptake and metabolize all added PROG, independently of being pre-treated with 24-EBL or not. Supporting this thought, PROG conjugates were found as a product of the conversion of exogenously applied PROG (Asthana and Asthana 2015). Considering this information, the obtained results imply that *S. nigrum* plants can absorb and metabolize this hormone without compromising their growth.

Photosynthesis is a primary physiological process. Currently, photosynthetic pigment contents are good criteria for assessing the effect of organic pollutants on the photosynthetic apparatus. As mentioned, no significant changes in the photosynthetic pigments’ levels were observed with the described treatments, indicating that both treatments had no adverse effect on this parameter. Regarding the action of 24-EBL, these findings are in agreement with those reported by earlier studies that showed that a pre-treatment with BR did not change photosynthetic pigment contents (Chl a, Chl b, and carotenoids) (Soares et al. 2016; Sousa et al. 2020b), implying that BRs are not capable of improving photosynthetic apparatus under non-stressful conditions.

Hydrogen peroxide can cause phytotoxic reactions such as lipid peroxidation and protein degradation. For this reason, MDA content, an indicator of membrane lipid peroxidation, is an excellent index to measure the toxic effects of pollutants (Soares et al. 2019). PROG exposure did not increase H_2_O_2_ levels. PROG alone significantly decreased both MDA and H_2_O_2_ levels in both organs studied. To date, there are only a few reports concerned with the effects of PROG on plant oxidative stress. A study with several mammalian sex hormones, including PROG, showed that a co-treatment with these hormones significantly ameliorated the adverse effects of plants exposed to salt stress. This effect was associated with a decrease in H_2_O_2_ and MDA levels (Erdal 2012). Under the present results, a previous report showed that the lower levels of ROS and lipid peroxidation in plants treated with PROG were due to an increase in the activity of ROS-scavenging enzymes such as SOD and CAT (Erdal and Dumlupinar 2011). Such results revealed more positive effects of PROG on the plant antioxidant system than oxidative damages. Accordingly, the biochemical data obtained in this study for total chlorophyll and carotenoids and a decrease in MDA and H_2_O_2_ levels strengthen the notion that *S. nigrum* plants did not suffer oxidative stress when exposed to PROG.

Likewise, the levels of H_2_O_2_ and lipid peroxidation on plants pre-treated with 24-EBL and exposed to PROG (Table 2) presented a decrease for the same biochemical parameters that was even more pronounced than with PROG alone, suggesting a synergistic effect of PROG and BR on *S. nigrum* plants’ ROS removal capacity. It is known that BRs can decrease lipid peroxidation even without any adverse situations (Sharma et al. 2015). This decrease in lipid peroxidation could be regarded as evidence for BR-induced efficient ROS scavenging mechanisms, like proline, which can scavenge ROS (Hayat et al. 2012) and which levels increased in both organs of the plants treated with the two hormones (see below). Therefore, significant reductions in H_2_O_2_ and lipid peroxidation levels observed in this study, more pronounced in plants from the PROG/24-EBL treatment, must result from an efficient ROS scavenging response synergistically induced by both hormones.

Proline is a proteinogenic amino acid that, under stressful conditions, acts like an osmoprotector and as a potent antioxidant, participating in the repair processes of membranes and ROS scavenging. Elevation in proline content has been reported under several types of stresses (de Sousa et al. 2013; Gill and Tuteja 2010; Martins et al. 2021; Sharma et al. 2015; Sousa et al. 2020a). In the present study, the increased proline levels in both organs of PROG/24-EBL-treated plants suggest that it may protect *S. nigrum* plants by acting as a scavenger of H_2_O_2_, thus contributing more to the lower MDA levels detected.

Nevertheless, the two tested treatments may induce other antioxidant defense mechanisms to decrease H_2_O_2_ and lipid peroxidation levels, such as AsA, thiols, or peroxidases (Moreira et al. 2018). In this study, the activity of several antioxidant enzymes and their relative transcript accumulation were studied. In PROG and PROG/24-EBL-treated plants, the reduction in H_2_O_2_ levels can also be related to the enhanced activity of the H_2_O_2_-scavenging enzyme APX. The increase observed in APX activity suggests that both treatments for both organs improved the ROS scavenging function of this enzyme. In accordance with these results, it was previously demonstrated that a pre-treatment with BR can increase APX activity in *Solanum lycopersicum* L. plants when exposed to polychlorinated biphenyls (Ahammed et al. 2013) and in *S. nigrum* plants exposed to Zn (Sousa et al. 2020b). Despite the increased APX activity, there was no correlation with the corresponding mRNA levels in most situations, suggesting that posttranscriptional and posttranslational regulations promoted this enzyme’s activity and stability. A similar regulation pattern was suggested for APX in *S. nigrum* leaves of Cd- or Ni-treated plants (Fidalgo et al. 2011; Soares et al. 2016). Nevertheless, further research should be pursued by native PAGE to identify potential APX isoenzymes responsible for the increased activities recorded.

The significant decrease in lipid peroxidation observed in this study could also be due to the effective removal of lipid peroxides by GST and/or a result of the protective actions of GSH. Interestingly, both treatments reduced the GSH levels in both organs analyzed. PROG-treated plants had a decrease in GST activity in both organs analyzed, which suggests that GSH may be protecting *S. nigrum* cells by acting as an antioxidant, removing ROS, and not so much involved in the detoxification processes (conjugation) of PROG. Supporting this hypothesis is that two pathways have been described for processing PROG by plant cells and that all final transformation products are posteriorly glycosylated and not glutathiolated (Asthana and Asthana 2015). Shoot GST was upregulated in response to BR in tomato (Ahammed et al. 2012) and, in the present study, the PROG/24-EBL treatment significantly increased shoot GST activity and its transcripts levels. Such increased activity presumably contributed to the observed reduction in GSH levels, thus suggesting a potential enhancement in the shoot cellular detoxification processes through the conjugation of GSH with potentially toxic products, like lipid peroxides, being activated by BR (Ahammed et al. 2012). Moreover, the correlation between mRNA levels and GST activities in shoots and roots from both treatments denotes that this enzyme was regulated by both hormones at the transcriptional level.

GSH is maintained in a predominantly reduced state by the enzyme GR (Soares et al. 2019). The increase recorded for GR activity in shoots of both treatments was essential to providing GSH to function as an antioxidant and protect photosynthesis. Supporting this observation, it was recently demonstrated that seedlings exposed to Pb derived from GSH-primed seeds registered elevated photosynthetic activity (Ahmad et al. 2023). However, no differences were detected regarding its mRNA accumulation, thus suggesting that this enzyme was regulated post-transcriptional and/or post-translationally, as already proposed for the GR in the bundle sheath cells of maize (Pastori et al. 2000).

The results obtained with the PROG/24-EBL treatment reveal that the increased shoot γ-ECS activity (the critical enzyme in the GSH biosynthesis process) was a response to the depletion in the GSH levels. Moreover, there was a strong correlation between the activity of γ-ECS and its encoding mRNA accumulation in all organs and situations analyzed. This suggests that γ-ECS was mainly regulated at its transcription by both hormones. γ-ECS primary regulation at its transcription has also been proposed for *S. nigrum* exposed to the end-products of the photo-Fenton process of metalaxyl (Teixeira et al. 2015) and to paracetamol (Martins and Teixeira 2021). Most importantly, in this study, there was an organ-dependent differential response to the PROG/24-EBL treatment, as the biosynthesis, reduction, and usage of GSH increased in shoots and decreased in roots. This can be explained by the fact that BR exogenously applied to shoots barely suffers translocation to other parts of the plant (Taiz and Zeiger 2010), thus exerting their beneficial effects mainly at the site of application, which in this case corresponds to the shoots, being the untreated parts of the plant (roots) depleted in the GSH-related biosynthesis necessary resources.

In conclusion, this study unequivocally establishes that *S. nigrum* plants can grow in PROG-contaminated sites without experiencing (oxidative) stress. Furthermore, it shows that the exogenous application of both steroid hormones (PROG and BR) increased the plants’ antioxidant response system, with γ-ECS and GST being directly controlled at the transcription level. At the same time, APX and GR were regulated post-transcriptionally. Additionally, it demonstrates that although these two steroidal hormones have distinct modes of action, both leading to a better performance against ROS when supplied simultaneously, they synergistically affect such performance and the removal of PROG from the environment.

## Data Availability

The datasets generated during and/or analyzed during the current study are available from the corresponding author upon reasonable request.
